# Mesenchymal Stem Cells Attenuate Radiation-Induced Brain Injury by Inhibiting Microglia Pyroptosis

**DOI:** 10.1155/2017/1948985

**Published:** 2017-12-07

**Authors:** Huan Liao, Hongxuan Wang, Xiaoming Rong, Enqin Li, Ren-He Xu, Ying Peng

**Affiliations:** ^1^Department of Neurology, Sun Yat-Sen Memorial Hospital, Sun Yat-Sen University, Guangzhou, China; ^2^Guangdong Provincial Key Laboratory of Malignant Tumor Epigenetics and Gene Regulation, Sun Yat-Sen Memorial Hospital, Sun Yat-Sen University, Guangzhou, China; ^3^Faculty of Health Sciences, University of Macau, Taipa, Macau

## Abstract

Radiation-induced brain injury (RI) commonly occurs in patients who received head and neck radiotherapy. However, the mechanism of RI remains unclear. We aimed to evaluate whether pyroptosis was involved in RI and the impact of mesenchymal stem cells (MSCs) on it. BALB/c male mice (6–8 weeks) were cranially irradiated (15 Gy), and MSCs were transplanted into the bilateral cortex 2 days later; then mice were sacrificed 1 month later. Meanwhile, irradiated BV-2 microglia cells (10 Gy) were cocultured with MSCs for 24 hours. We observed that irradiated mice brains presented NLRP3 and caspase-1 activation. RT-PCR then indicated that it mainly occurred in microglia cells but not in neurons. Further, irradiated BV-2 cells showed pyroptosis and increased production of IL-18 and IL-1*β*. RT-PCR also demonstrated an increased expression of several inflammasome genes in irradiated BV-2 cells, including NLRP3 and AIM2. Particularly, NLRP3 was activated. Knockdown of NLRP3 resulted in decreased LDH release. Noteworthily, in vivo, MSCs transplantation alleviated radiation-induced NLRP3 and caspase-1 activation. Moreover, in vitro, MSCs could decrease caspase-1 dependent pyroptosis, NLRP3 inflammasome activation, and ROS production induced by radiation. Thus, our findings proved that microglia pyroptosis occurred in RI. MSCs may act as a potent therapeutic tool in attenuating pyroptosis.

## 1. Introduction

Radiotherapy is commonly used in patients with head and neck cancer [1]. However, radiation can inevitably damage the adjacent normal brain tissues [2, 3] and induce late-onset brain injury [4–7].

At present, several theories have been proposed to explain the development of radiation-induced brain injury, including direct injury from radiation [8–10], damage on cerebral vascular system [11, 12], immunoinflammatory responses [13], and oxidative stress [14]. However, corresponding therapies to those theories including using anti-inflammatory agents have not shown enough efficacy in human patients. Therefore, the underlying mechanism by which radiation induces inflammatory response has not yet been completely elucidated.

Pyroptosis, a regulated cell death, is dependent on caspase-1 activation, which eventually causes the loss of plasma membrane integrity [15–17] as well as promoting inflammatory response [18]. Previous studies found that the development of pyroptosis has a close relationship with the activation of inflammasomes [16]. Among whole inflammasomes, NLRP3 (NLR pyrin domain containing 3) inflammasome is the most studied. The activation of NLRP3 inflammasome, which includes increase of NLRP3 at protein level and assembly of ASC and pro-caspase-1, results in active caspase-1. Recent studies indicate that caspase-1 and inflammasomes expression were significantly upregulated in the lung tissue and oral mucosa after radiation [19, 20]. However, whether pyroptosis occurs in radiation-induced brain injury is still unclear.

Mesenchymal stem cells (MSCs) have emerged as promising cell therapies for multiple diseases based on demonstrations of their potent immunomodulatory capacities [21, 22]. Previous studies found that MSCs transplantation could improve cognition of rats exposed to radiation [23, 24]. Moreover, MSCs were reported to be able to inhibit NLRP3 inflammasome activation [25]. Whether MSCs alleviate radiation-induced brain injury by inhibiting NLRP3 inflammasome activation is still unknown.

This study aimed to investigate whether pyroptosis occurred in radiation-induced brain injury (RI) and the role of MSCs in treating RI especially on inhibiting pyroptosis.

## 2. Materials and Methods

### 2.1. Cell Culture and Irradiation

BALB/c male newborn mice (2-3 days) were purchased from the Laboratory Animal Center of South Medical University. All animal procedures followed the humane care guidelines of the Chinese National Institute of Health, and the protocols were approved by the Committee on Animal Research of Sun Yat-Sen University. For primary neurons culture, the meninges, brain stem, and cerebellum of pups were removed after being sterilized with 70% ethanol. The remaining tissue was cut into small pieces, washed with serum-free Dulbecco's Modified Eagle Medium (DMEM) (Gibco), and then digested with a mix of Dnase I (1 KU/ml, Sigma) and Trypsin (0.25%, BIOSHARP) for 30 min at 37°C. After being spun down, the suspended cells in Neurobasal-A medium (Gibco) with 10% fetal bovine serum (FBS, Gibco), 2% B27 Supplement (Gibco), and 1% L-Glutamine (Sigma) were transferred to a T75 flask. After 6 h, the medium was changed into Neurobasal-A medium with 2% B27 Supplement and 1% L-Glutamine.

The immortalized murine microglia BV-2 cell line was obtained from the Cell Center of Peking Union Medical College in China. BV-2 cells were cultured in DMEM supplemented with 10% FBS and 1% penicillin/streptomycin (Gibco) at 37°C in a humidified incubator with 5% CO_2_. Both neurons and BV-2 cells were irradiated using a linear accelerator (Siemens, German). A single dose of 10 Gy was given at a rate of 3 MeV/min [26].

Human trophoblast-derived mesenchymal stem cells (MSCs) labeled with green fluorescence were gifted from Professor Ren-He Xu from University of Macau. Approval for using MSCs was obtained from the Animal Ethics Committee of Sun Yat-Sen Memorial Hospital. MSCs were grown in culture flasks at 10^5^ cells/cm^2^, in *α*–MEM (Gibco), supplemented with 10% heat-inactivated FBS, 1% L-Glutamine (Sigma), 1% penicillin/streptomycin, and 1% NEAA (Gibco). Passage 6 or passage 7 of MSCs was used for injection and coculture experiments.

### 2.2. MSCs and BV-2 Cells Coculture

Irradiated BV-2 microglia cells (10 Gy) were applied for coculture model with mesenchymal stem cells (MSCs) for 24 hours. MSCs were allowed to adhere before coculture with BV-2 cells in DMEM complete medium. BV-2 : MSCs coculture experiments were carried out as previous literature mentioned [27] (with slight modification). Briefly, BV-2 (in plate wells) : MSCs (in inserts) coculture experiments were carried out in 12-well plates at BV-2 : MSCs of 2 : 1; BV-2 = 2*∗*10^5^ cells.

### 2.3. Animal Irradiation and Treatment

BALB/c male mice (6–8 weeks) were purchased from the Laboratory Animal Center of South Medical University. Mice were randomly divided into six groups: (1) unirradiated controls, (2) unirradiated mice injected with MSCs, (3) unirradiated mice injected with PBS, (4) irradiated mice, (5) irradiated mice injected with MSCs, and (6) irradiated mice given PBS. Irradiation was administered using a 6 MV *β*-ionizing-ray linear accelerator (Siemens, Germany). The head of each mouse was placed in a treatment field (2*∗*2 cm^2^) within the confines of the whole brain from the postaurem line to the postcanthus line. A single dose of 15 Gy was given at a rate of 3 Gy/min.

At the second day postirradiation, each mouse in group 2 and group 5 (mentioned above) received bilateral, cortex transplantation of MSCs suspended in vehicle (PBS) using a 33-gauge microsyringe at an injection rate of 0.25 uL/min. Each cortex received two distinct injections of MSCs (1.0 × 10^5^ in 1 *μ*l) per hemisphere using precise stereotaxic coordinates, as described in previous research [28, 29]. Group 3 and group 6 (mentioned above) received equal volumes of sterile vehicle at the same stereotaxic coordinates. Animals were anesthetized with 4% chloral hydrate (500 mg/kg). All animals were sacrificed one month after transplantation.

### 2.4. Lactate Dehydrogenase Release Assay

After the cells had been exposed to the different treatments, the culture supernatant was harvested, and the LDH level was tested using the LDH cytotoxicity detection kit (Promega) according to the manufacturer's instructions.

### 2.5. Quantitative Real-Time PCR

Total mRNA and quantitative real-time PCR (q-PCR) were performed according to the classic protocol. We used real-time PCR to detect and quantify the expression of genes related to pyroptosis. The data were analyzed by Light Cycler 480 real-time PCR machine (Roche Applied Science).

### 2.6. Western Blotting Analysis

Total proteins were extracted using RIPA lysis buffer (Beyotime). The samples were continuously processed for western blotting as previous study described [30]. In brief, protein lysates were separated by SDS-PAGE and then electrophoretically transferred to polyvinyl difluoride membrane. After being blocked in 5% nonfat milk, the blots were laid on a shaker and incubated with caspase-1 (1 : 100, Sanra Cruz), NLRP3 (2 *μ*g/ml, Thermo Fisher Scientific), and *β*-actin (1 : 1000, CST) overnight at 4°C. After washing with TBST, the membranes were probed with HRP- (horseradish peroxidase-) conjugated secondary antibody (1 : 5000, Multi Sciences). Finally, the blots developed by enhanced chemiluminescence detection (ECL, KeyGen Biotech).

### 2.7. Measurement of Intracellular ROS Levels

Using a Reactive Oxygen Species Assay Kit (Beyotime) according to the manufacturer's protocols, the production of intracellular ROS was measured. After the proper treatments, the fluorescence was measured with a microplate reader (Tecan).

### 2.8. Enzyme-Linked Immunosorbent Assay

ELISAs were performed to measure levels of IL-1*β* and IL-18 on the culture supernatants according to the manufacturer's instructions (eBioscience).

### 2.9. Small Interfering RNA Transfections

NLRP3 siRNA and negative control (NC) were purchased from RiboBio. BV-2 cells were transfected using the transfection reagent Lipofectamine 2000 (Invitrogen) for 6 hours. Medium was then changed and cells continued to be incubated for 24 hours before subsequent experiments.

### 2.10. Immunofluorescence

The BV-2 microglia cells from 12-well were fixed with 4% paraformaldehyde (PFA), permeabilized with 0.1% Triton X-100, and then blocked with 1% BSA. Afterward, the permeabilized cells were incubated at 4°C overnight with primary antibodies against caspase-1 (1 : 50). After washing, the cells were stained with Alexa Fluor 488 second antibody (1 : 2000, Beyotime). After nuclear staining with DAPI (1 : 1000, Sigma), samples were observed and photographed with a laser scanning confocal microscopy (LSCM).

Mice brains slices were sectioned with thickness of 20 *μ*m. For the detection of NLRP3, mice brain slices were processed as mentioned above with anti-NLRP3 (20 *μ*g/ml, Thermo Fisher Scientific). Finally, the slices were observed with a fluorescence microscope.

### 2.11. Statistical Analysis

Quantitative data was expressed as mean ± SEM. Data were compared using one-way analysis of variance or Student's *t*-test. *p* < 0.05 was considered statistically significant for all comparisons. All statistical analyses were performed using SPSS statistical package (version 22.0 for Windows, SPSS Inc., USA).

## 3. Results

### 3.1. Radiation Increases NLRP3 Inflammasome and Caspase-1 p20 Expression in the Brain

**Figure 1:**
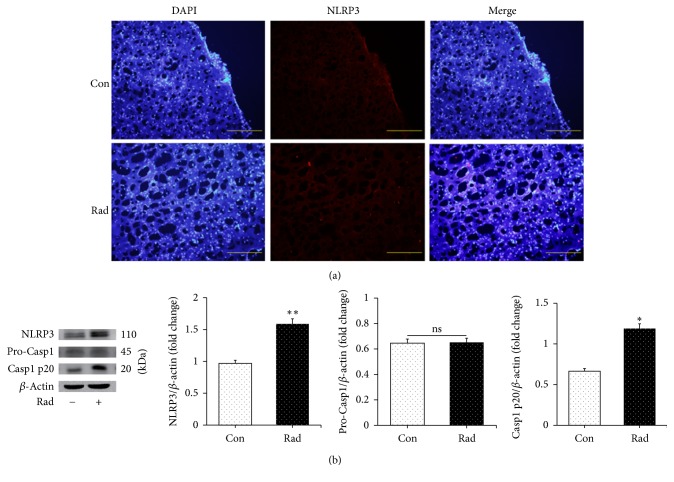
Radiation increased cortex NLRP3 inflammasome and caspase-1 expression in vivo. (a) Immunofluorescence assay proved that increased NLRP3 inflammasome expression was induced by radiation in vivo. (b) Western blotting analysis demonstrated that mice brains exposed to 15 Gy radiation had increased expression of NLRP3 inflammasome and caspase-1. Bars represent mean** ±** SEM. *n* = 3~7. Scale bar indicates 50 *μ*m. ^*∗*^*p* < 0.05 and ^*∗∗*^*p* < 0.01 versus the control group (con); ns means no significance.

To determine whether increased NLRP3 and caspase-1 expression is present in radiation-induced brain injury, we examined the cortices from the mice that had received 15 Gy irradiation. Mice were sacrificed one month after treatment and the cortices were removed for analysis. Immunofluorescence assay showed significant increased NLRP3 inflammasome expression after radiation compared with those without radiation (Figure 1(a)). Additionally, western blot demonstrated that radiation significantly increased protein NLRP3 and caspase-1 p20 expression when compared with that in the control group (Figure 1(b)). These data indicate that radiation can increase cortex NLRP3 and caspase-1 expression in vivo.

### 3.2. Radiation Provokes Microglia Pyroptosis

**Figure 2:**
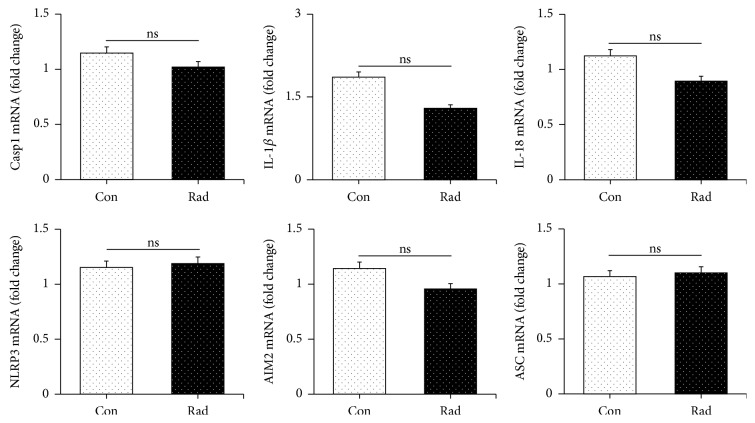
The mRNA expression of pyroptosis-related genes showed no significant difference in neurons between radiation group and control group. Bars represent mean ± SEM. *n* = 3~7; ns means no significance.

**Figure 3:**
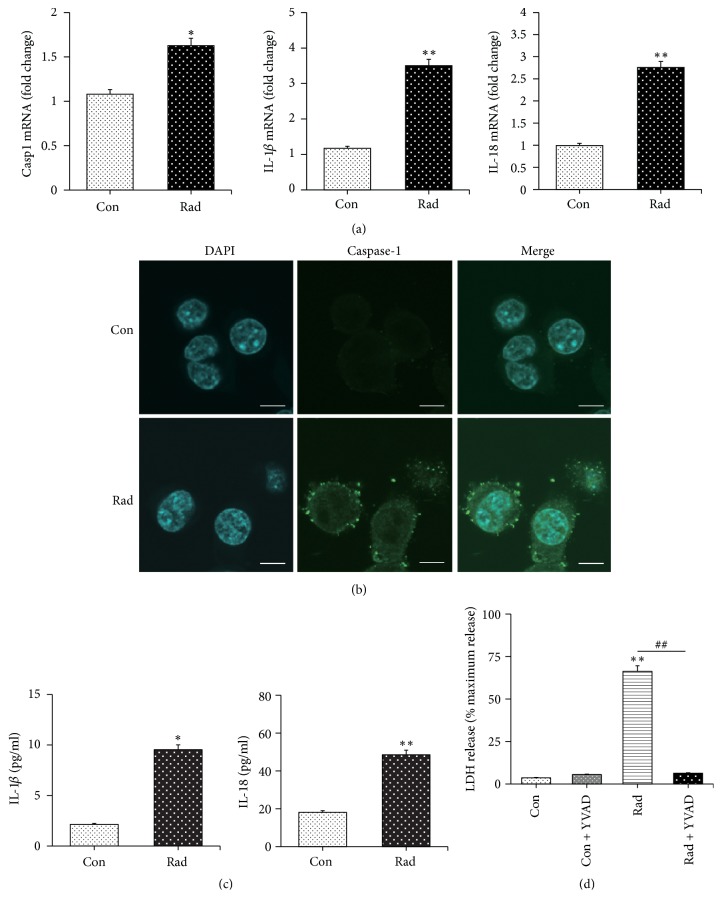
Radiation induced caspase-1-dependent pyroptosis in microglia and increased IL-18 and IL-1*β* production. (a) 10 Gy radiation promoted the expression of caspase-1 as well as proinflammation cytokine including IL-18 and IL-1*β* in BV-2 microglia cells at the transcriptional level. (b) Data from laser scanning confocal microscopy demonstrated caspase-1 activation in BV-2 microglia cells exposed to radiation. (c) Increased IL-18 and IL-1*β* experienced upregulating after radiation. (d) LDH production increased after radiation exposure in BV-2 microglia cells while experiencing decrease after the caspase-1 inhibitor Ac-YVAD was treated. Bars represent mean** ±** SEM. *n* = 3~7. Scale bar indicates 10 *μ*m. ^*∗*^*p* < 0.05 and ^*∗∗*^*p* < 0.01 versus the control group (con); ^##^*p* < 0.01 versus the Rad group.

To determine what type of cells developed pyroptosis, we examined both neurons and microglia by using primary neurons and BV-2 cells in vitro, respectively. As quantitative RT-PCR showed, the mRNA expression of caspase-1, IL-1*β*, IL-18, NLRP3, and AIM2 as well as ASC had no significant difference in neurons between radiation group and control group (Figure 2), while, in BV-2 cells, radiation increased the mRNA expression of caspase-1, IL-1*β*, and IL-18 (Figure 3(a)). Additionally, as shown in Figure 3, radiation increased the protein expression of active caspase-1 apparently (Figure 3(b)). We also found that the expression of IL-1*β* and IL-18 increased significantly, especially the amount of IL-18 with nearly sevenfold change by ELISA (Figure 3(c)).

According to previous studies [15–17], pyroptosis leads to membrane pore formation and cell lysis, and LDH will then be released. Consistently, our results showed that BV-2 microglia cells in radiation group released more LDH when compared with those in control group. Caspase-1 inhibitor Ac-Tyr-Val-Ala-Asp-chloromethylketone (Ac-YVAD, Sigma-Aldrich, added to cells at 10 *μ*g/mL at least 30 mins before radiation [27]) could reduce the secretion of LDH (Figure 3(d)). Here, our data proved that radiation could induce pyroptosis of BV-2 microglia.

### 3.3. Radiation Activated NLRP3 Which May Be Involved in BV-2 Microglia Pyroptosis

**Figure 4:**
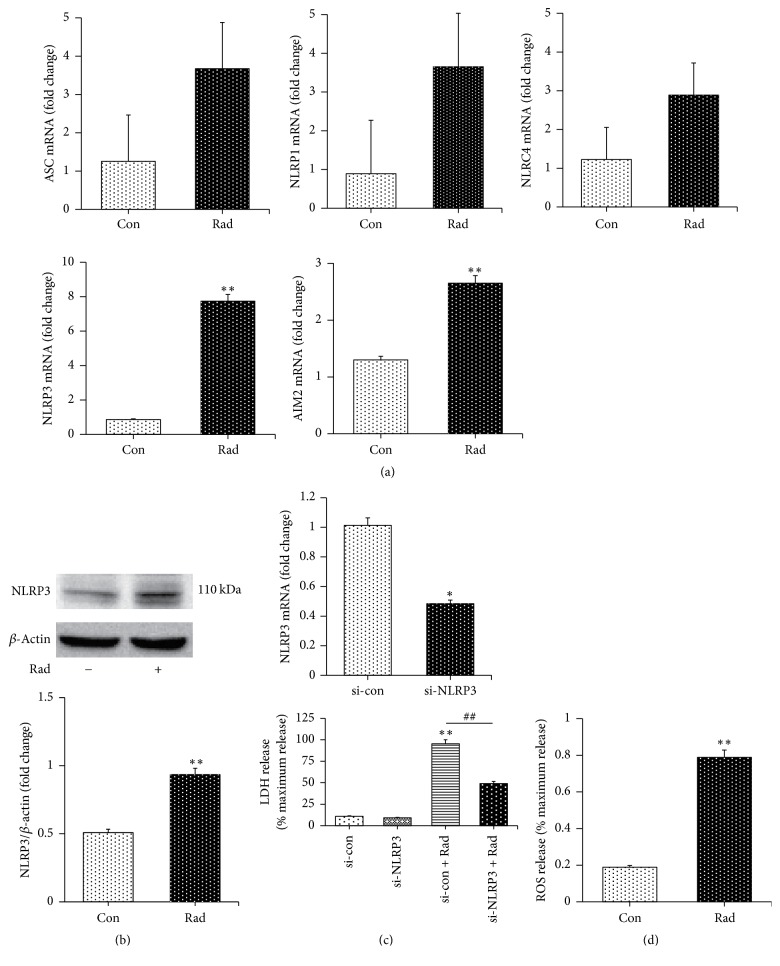
Radiation activated several inflammasomes including NLRP3 which might have taken part in pyroptosis in BV-2 microglia cells exposed to radiation with promoted ROS production. (a) Radiation promoted both AIM2 and NLRP3 increase significantly as well as increasing the expression of ASC, NLRP1, and NLRC4 with no significance. (b) Radiation induced NLRP3 inflammasome activation. (c) After knockdown of NLRP3, LDH production decreased. (d) ROS production increased after radiation. Bars represent mean ± SEM. *n* = 3~7. ^*∗*^*p* < 0.05 and ^*∗∗*^*p* < 0.01 versus the control group (con); ^##^*p* < 0.01 versus the Rad group.

Additionally, we found that radiation promoted the expression of some inflammasomes at the transcriptional level, including ASC inflammasome, NLRP1 inflammasome, NLRC4 inflammasome, NLRP3 inflammasome, and AIM2 inflammasome. Of note, NLRP3 inflammasome increased most obviously, with almost eightfold change (Figure 4(a)). Similarly, radiation could activate NLRP3 in BV-2 cells (Figure 4(b)). By knocking down the NLRP3 gene expression by siRNA, we found that the LDH release was decreased (Figure 4(c)), which indicated the role of NLRP3 in radiation-induced pyroptosis in BV-2 microglia cells. Moreover, considering that ROS production is one of the mechanisms involved in NLRP3 inflammasome activation [31], we also measured the level of ROS production of BV-2 cells after radiation, and the data showed that radiation significantly increased ROS generation (Figure 4(d)). Here, our results demonstrated that radiation activated NLRP3 which might be involved in BV-2 microglia pyroptosis.

### 3.4. MSCs Inhibit Radiation-Induced Pyroptosis

**Figure 5:**
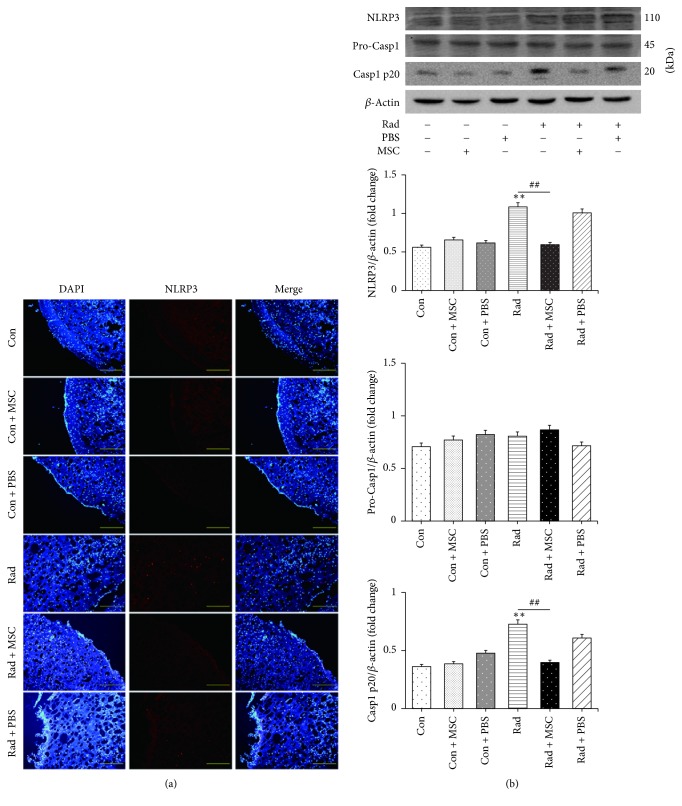
MSCs alleviated the activation of NLRP3 inflammasome and caspase-1 caused by radiation in vivo. (a) Immunofluorescence assay proved that MSCs prevented the NLRP3 inflammasome activation induced by radiation in vivo. (b) Western blotting analysis demonstrated that mice brains exposed to 15 Gy radiation experienced activation of NLRP3 inflammasome and caspase-1 which was successfully prevented by MSCs transplantation through stereotactic intracranial injection a month later. Scale bar indicates 50 *μ*m. Bars represent mean** ±** SEM. *n* = 3~7, ^*∗∗*^*p* < 0.01 versus the control group (con); ^##^*p* < 0.01 versus the Rad group.

**Figure 6:**
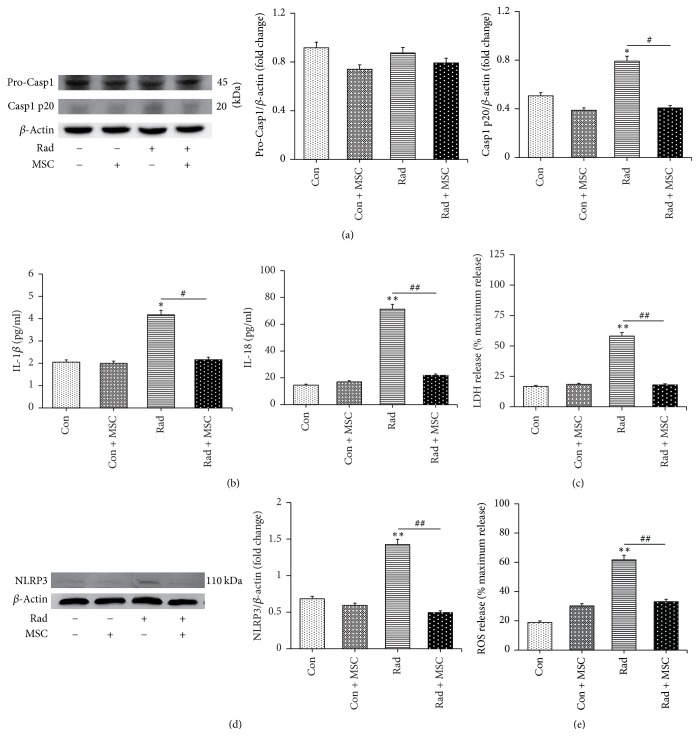
Radiation-induced caspase-1-dependent pyroptosis, increased IL-18 and IL-1*β* production, and NLRP3 inflammasome activation in microglia were prevented by MSCs. (a) MSCs decreased caspase-1 activation in BV-2 microglia cells exposed to radiation. (b) Increased IL-18 and IL-1*β* experienced downregulating when coculture was performed after radiation. (c) Increased LDH production in BV-2 microglia cells experienced decrease after MSCs were treated. (d) Radiation-induced NLRP3 inflammasome activation was prevented by MSCs. (e) MSCs prevented ROS production induced by radiation. Bars represent mean ± SEM. *n* = 3~7. ^*∗*^*p* < 0.05 and ^*∗∗*^*p* < 0.01 versus the control group (con); ^#^*p* < 0.05 and ^##^*p* < 0.01 versus the Rad group.

In an attempt to establish a functional role for MSCs in radiation-induced pyroptosis, we utilized a radiation animal model and implanted MSCs into the BALB/c mice two days after radiation. Immunofluorescence assay and western blotting showed that the brain cortex with MSCs transplantation had less NLRP3 and caspase-1 activation compared with the control group (Figures 5(a) and 5(b)). Similarly, in an in vitro study, BV2 cells cocultured with MSCs showed less caspase-1 activation (Figure 6(a)), less IL-1*β* and IL-18 secretion (Figure 6(b)), less LDH (Figure 6(c)), and less NLRP3 inflammasome activation (Figure 6(d)) as well as ROS release (Figure 6(e)) when compared with the cells without coculture. Altogether, our data indicated that MSCs have potent ability to alleviate radiation-induced pyroptosis by inhibiting NLRP3 and caspase-1 activation.

## 4. Discussion

Our study firstly demonstrated that microglia induced pyroptosis and NLRP3 inflammasome activation in late-onset RI which can be prevented by mesenchymal stem cells.

Firstly, our findings proved that radiation not only induced inflammasome NLRP3 but also elicited caspase-1 activation in mice brain cortices. Next, we verified that BV-2 microglia cells exposed to radiation experienced caspase-1 activation, with increased LDH which was prevented by caspase-1 inhibitor and increased IL-18 and IL-1*β* secretion, indicating pyroptosis developed. Moreover, our data revealed radiation activated several inflammasomes including NLRP3 which might be involved in BV-2 microglia pyroptosis with promoted ROS release. What is more, MSCs transplantation proved to be an effective way to reduce NLRP3 and caspase-1 activation and the data of coculture implied further that MSCs could serve as a therapeutic way to cure microglia pyroptosis. As well, ROS production from BV-2 microglia cells exposed to radiation was reduced by MSCs.

Previous studies suggested that RI not only was related to immune system reaction [26, 32], such as promoting proinflammation factors expression, which was also involved in necrosis, but also had close relationship with cell death, such as apoptosis [33]. Given the fact that pyroptosis possesses the characteristics of both apoptosis and necrosis [15], our data showing pyroptosis induced by inflammasome activation and inflammatory response in microglia induced by radiation furtherly enhanced the understanding of the above characteristics. There are some researches suggesting that radiation exposure can cause inflammasome activation and caspase-1 activation in some kinds of cells and tissues, such as immune cells [34] and lung tissues [20], which is supportive of our findings. To the best of our knowledge, this is the first time to report that RI is related to microglia pyroptosis and NLRP3 inflammasome activation. Meantime, we demonstrated that several inflammasomes were activated in BV-2 microglia cells exposed to radiation, especially AIM2 and NLRP3 significantly, which indicated that microglia pyroptosis occurred after radiation exposure was not dependent on only one inflammasome but more. Moreover, as previous researches mentioned, NLRP3 inflammasome, as an intracellular heteromeric complex expressed in immune cells, is made up of the NLRP3 protein, apoptosis-associated speck-like protein containing the caspase recruitment domain (ASC) and pro-caspase-1 [35]. After the activation and assembly of NLRP3 inflammasome, pro-caspase-1 is cleaved via autocatalytic processes to transform into the active form of caspase-1, which comprises a tetramer containing two 20 kD fragments (Casp1 p20) and two 10 kD fragments, which process the maturation of pro-interleukin (pro-IL-1*β*) and pro-IL-18 cytokines into their active and secreted forms, IL-1*β* and IL-18 [18]. The result of knockdown of NLRP3 in our study implied that NLRP3 inflammasome activation may have played a dominated role in microglia pyroptosis involved in RI, while whether there exists some other inflammasomes involved in microglia pyroptosis needs more investigation. Furthermore, we confirmed that increased ROS release happened in BV-2 microglia cells exposed to radiation, which was consistent with researches done before [14].

Nowadays, glucocorticoids are the main therapeutical way to alleviate the symptoms of RI [36], which actually have not got satisfaction from most patients. This grim reality urged us to develop new treatments for curing RI. MSCs show their potential abilities to cure various untreatable diseases [37] and brain injury [38]. Noticeably, MSCs transplantation could improve cognition of rats exposed to radiation [23, 24]. However, they did not investigate deeper mechanism. Our findings proved that MSCs transplantation could ameliorate pyroptosis and NLRP3 inflammasome activation caused by radiation, which offered a new way to explain how MSCs acted as a potent therapy from the aspect of mechanism. In addition, we can assume that alleviating pyroptosis and NLRP3 inflammasome activation in the brain is one important mechanism of improving cognition of radiation exposed rats. What is more, we can even speculate that MSCs enable improving other disorders in central nervous system and even in other body systems involving pyroptosis and NLRP3 inflammasome activation. Additionally, we found that MSCs could decrease ROS production. Previous studies suggested that ROS played a critical role in inflammasome activation, especially NLRP3 inflammasome [32]. In our study, NLRP3 knockdown decreased LDH production. These facts drop us a hint that MSCs enable diminishing inflammasome activation through decreasing ROS production, which can further alleviate diseases involving inflammasome activation. Besides, ROS is closely associated with the development of all kinds of diseases [39], so MSCs offer a novel way to treat diseases involving ROS release.

## 5. Conclusions

Altogether, our study firstly implied that the mechanism of RI involves pyroptosis, including microglia pyroptosis which may be dependent on NLRP3 inflammasome activation. And MSCs offer a novel therapeutical way to radiation-induced brain injury via alleviating pyroptosis and decreasing ROS production. These findings might be applied to other diseases caused by radiation and illness involving pyroptosis and ROS production increasing. Further researches on the molecular pathways involved in MSCs for preventing pyroptosis in cells are imperative to improve MSC-based therapeutic settings in radiation-induced diseases and in regenerative medicine.
