# Corrigendum to “Mesenchymal Stem Cells Attenuate Radiation-Induced Brain Injury by Inhibiting Microglia Pyroptosis”

**DOI:** 10.1155/2018/1965958

**Published:** 2018-07-18

**Authors:** Huan Liao, Hongxuan Wang, Xiaoming Rong, Enqin Li, Ren-He Xu, Ying Peng

**Affiliations:** ^1^Department of Neurology, Sun Yat-Sen Memorial Hospital, Sun Yat-Sen University, Guangzhou, China; ^2^Guangdong Provincial Key Laboratory of Malignant Tumor Epigenetics and Gene Regulation, Sun Yat-Sen Memorial Hospital, Sun Yat-Sen University, Guangzhou, China; ^3^Faculty of Health Sciences, University of Macau, Taipa, Macau, China

In the article titled “Mesenchymal Stem Cells Attenuate Radiation-Induced Brain Injury by Inhibiting Microglia Pyroptosis” [1], Dr. Enqin Li and Dr. Ren-He Xu were missing from the authors' list. They contributed to establishing the mesenchymal stem cell line and maintaining the quality of the cell cultures. They worked on the stem cells for the subsequent experiment. The corrected authors' list is shown above and corrected in place.

Additionally, the sentence “Thanks are due to Professor Ren-He Xu from University of Macau for his gifts of human trophoblast-derived mesenchymal stem cells” should be removed from the Acknowledgments. This has been corrected in place.
